# Author Correction: The transcriptional repressor complex FRS7-FRS12 regulates flowering time and growth in *Arabidopsis*

**DOI:** 10.1038/ncomms16213

**Published:** 2018-04-10

**Authors:** Andrés Ritter, Sabrina Iñigo, Patricia Fernández-Calvo, Ken S. Heyndrickx, Stijn Dhondt, Hua Shi, Liesbeth De Milde, Robin Vanden Bossche, Rebecca De Clercq, Dominique Eeckhout, Mily Ron, David E. Somers, Dirk Inzé, Kris Gevaert, Geert De Jaeger, Klaas Vandepoele, Laurens Pauwels, Alain Goossens

Nature Communications 8: Article number: 15235; DOI: 10.1038/ncomms15235 (2017); Published online: 05 11 2017; Updated: 04 10 2018

The financial support for this Article was not correctly acknowledged. The Acknowledgements incorrectly stated that the study was funded in part by Research Foundation Flanders for project GN005212.

The Acknowledgements should have stated that the study was funded in part by Research Foundation Flanders for projects G005312N and G005915N.

This error has been corrected in both the PDF and the HTML versions of the Article.

