# Supplementary material for: The transcriptional repressor complex FRS7-FRS12 regulates flowering time and growth in Arabidopsis
Source: Nat Commun. 2017 May 11;8:15235. doi: 10.1038/ncomms15235 (PMC5437275; doi:10.1038/ncomms15235)
Supplement: Supplementary Information — Supplementary Figures and Supplementary Tables [file ncomms15235-s1.pdf]

Supplementary Information

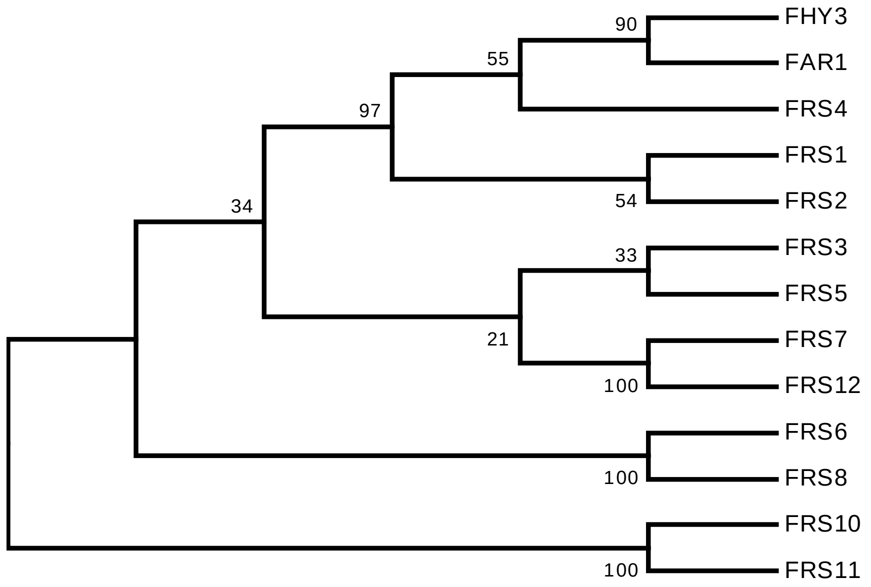

Supplementary Figure 1. The FRS family. Maximum-likelihood phylogenetic tree of the FAR1 RELATED SEQUENCE (FRS) family. Numbers above the branches represent bootstrap values.

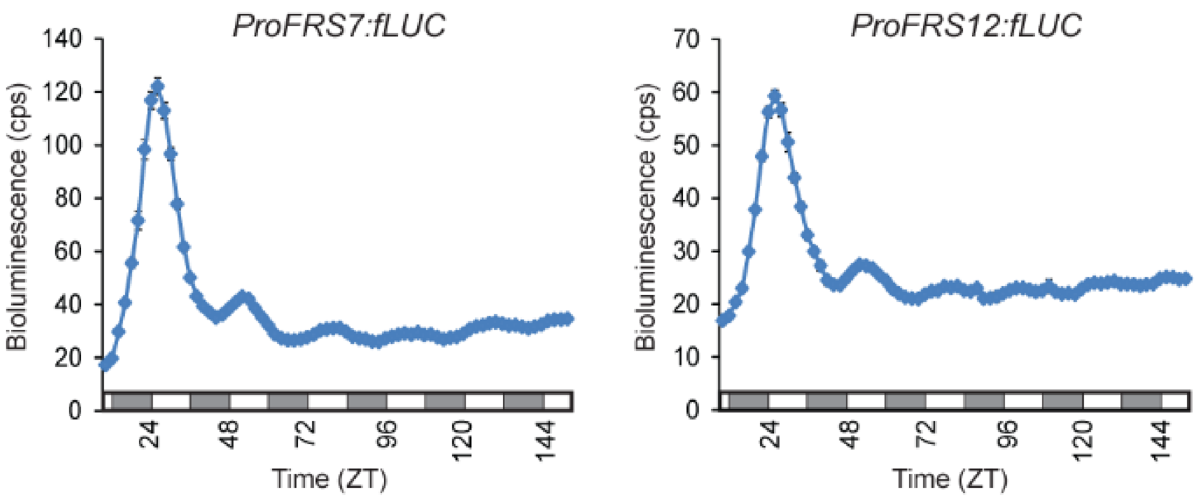

Supplementary Figure 2. Biological repeat of the circadian bioluminescence expression analysis of *FRS7* and *FRS12*. The experiment was conducted as described in the legend of Fig. 1a. Data represent the mean ± SEM (n = 6, corresponding to 6 wells of protoplasts that were imaged and averaged for each time point of the figure). White and gray regions indicate subjective light and dark period, respectively.

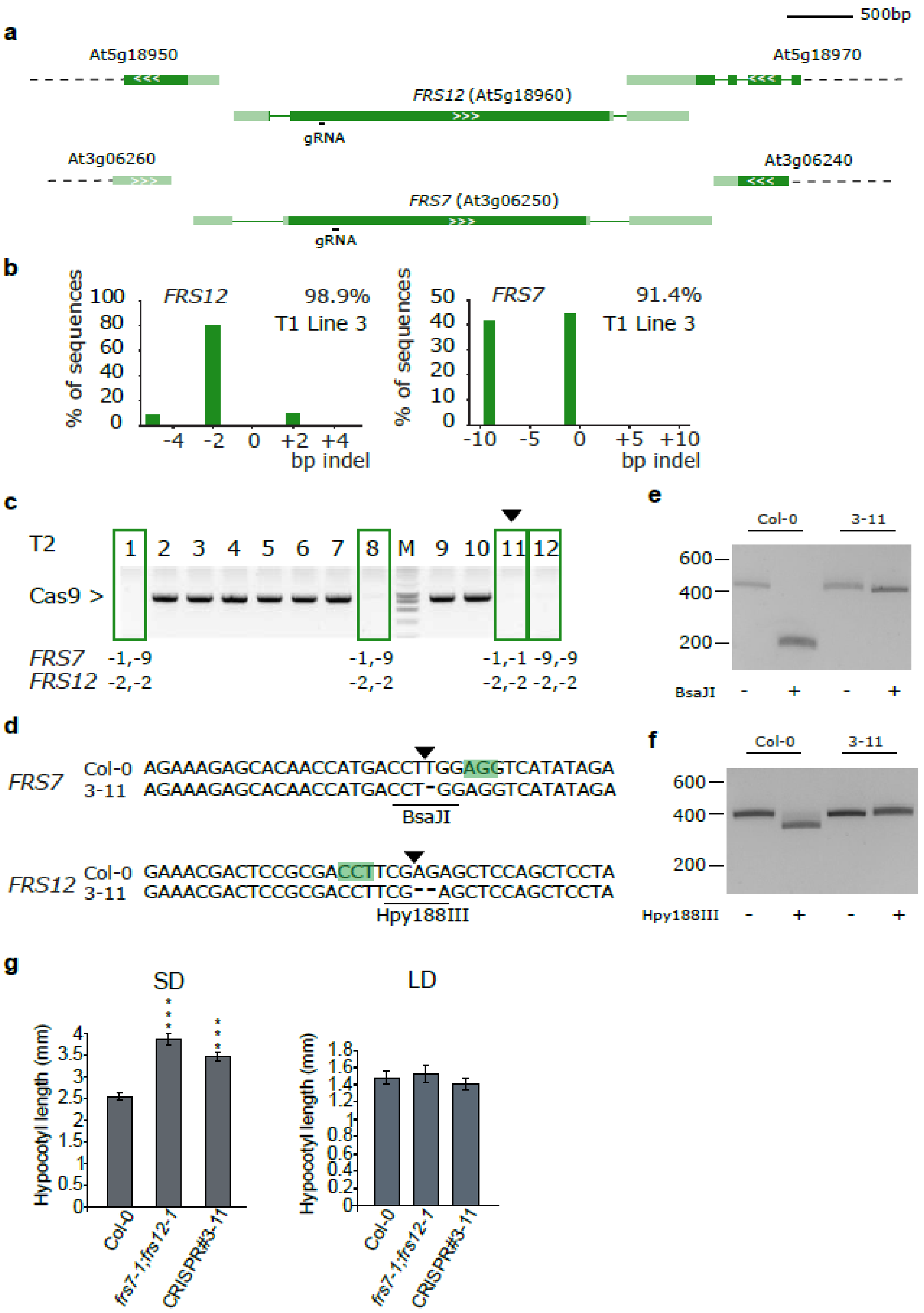
a
500bp
At5g18950
At5g18970
FRS12 (At5g18960)
gRNA
At3g06260
At3g06240
FRS7 (At3g06250)
gRNA
b
% of sequences
FRS12
98.9%
T1 Line 3
100
80
60
40
20
0
-4 -2 0 +2 +4
bp indel
FRS7
91.4%
T1 Line 3
50
40
30
20
10
0
-10 -5 0 +5 +10
bp indel
c
T2
1 2 3 4 5 6 7 8 M 9 10 11 12
Cas9 >
FRS7 -1,-9 -1,-9 -1,-1 -9,-9
FRS12 -2,-2 -2,-2 -2,-2 -2,-2
d
FRS7 Col-0 AGAAAGAGCACAACCATGACCTTGGAGGTCATATAGA
3-11 AGAAAGAGCACAACCATGACCT-GGAGGTCATATAGA
BsaJI
FRS12 Col-0 GAAACGACTCCGCGACCTTCGAGAGCTCCAGCTCCTA
3-11 GAAACGACTCCGCGACCTTCG--AGCTCCAGCTCCTA
Hpy188III
e
Col-0
3-11
600
400
200
BsaJI - + - +
f
Col-0
3-11
600
400
200
Hpy188III - + - +
g
SD
LD
Hypocotyl length (mm)
4
3.5
3
2.5
2
1.5
1
0.5
0
Col-0
frs7-1,frs12-1
CRISPR#3-11
1.8
1.6
1.4
1.2
1
0.8
0.6
0.4
0.2
0

Supplementary Figure 3. CRISPR/Cas9-mediated knock-out of *FRS7* and *FRS12*. a, genomic structure of the targeted genes and location of the sgRNAs. Dark green boxes designate exons; light green boxes, UTRs; solid lines, introns; arrows, gene orientation. **b**, TIDE analysis. Genomic DNA of a chimeric T1 plant (Line 3) was PCR amplified, sequenced by standard capillary electrophoresis and analyzed using TIDE. The indel spectrum is visualized with an estimated overall efficiency and frequency of each indel. **c**, PCR amplification of the Cas9 transgene. Null segregants are boxed and the continued plant marked with a triangle. TIDE estimated genotypes for *FRS7* and *FRS12* are given for the null segregants. **d**, Sequence alignment of the targeted loci for Col-0 and (Line 3, plant 11). PAM is highlighted and the Cas9 cut site is indicated with a triangle. Deleted bases are replaced by a dash. Restriction enzyme recognition sites overlapping the Cas9 cut site are underlined. **e and f**, Cleaved Amplified Polymorphic Sequences (CAPS) assay. Genomic DNA of Col-0 and T3 CRISPR #3-11 plants was used to amplify the genomic region spanning the mutation. PCR products were subsequently digested with the indicated restriction enzymes or mock digested. **g**, Hypocotyl length measurements of *Arabidopsis* Col-0 wt seedlings compared to loss-of-function *FRS7 FRS12* T-DNA line and T3 CRISPR #3-11 line. Seedlings were grown for 10 days under SD or LD conditions. Values represent the average of at least 24 biological replicates ± SEM in SD and 15 in LD conditions (****P*<0.001, t-test).

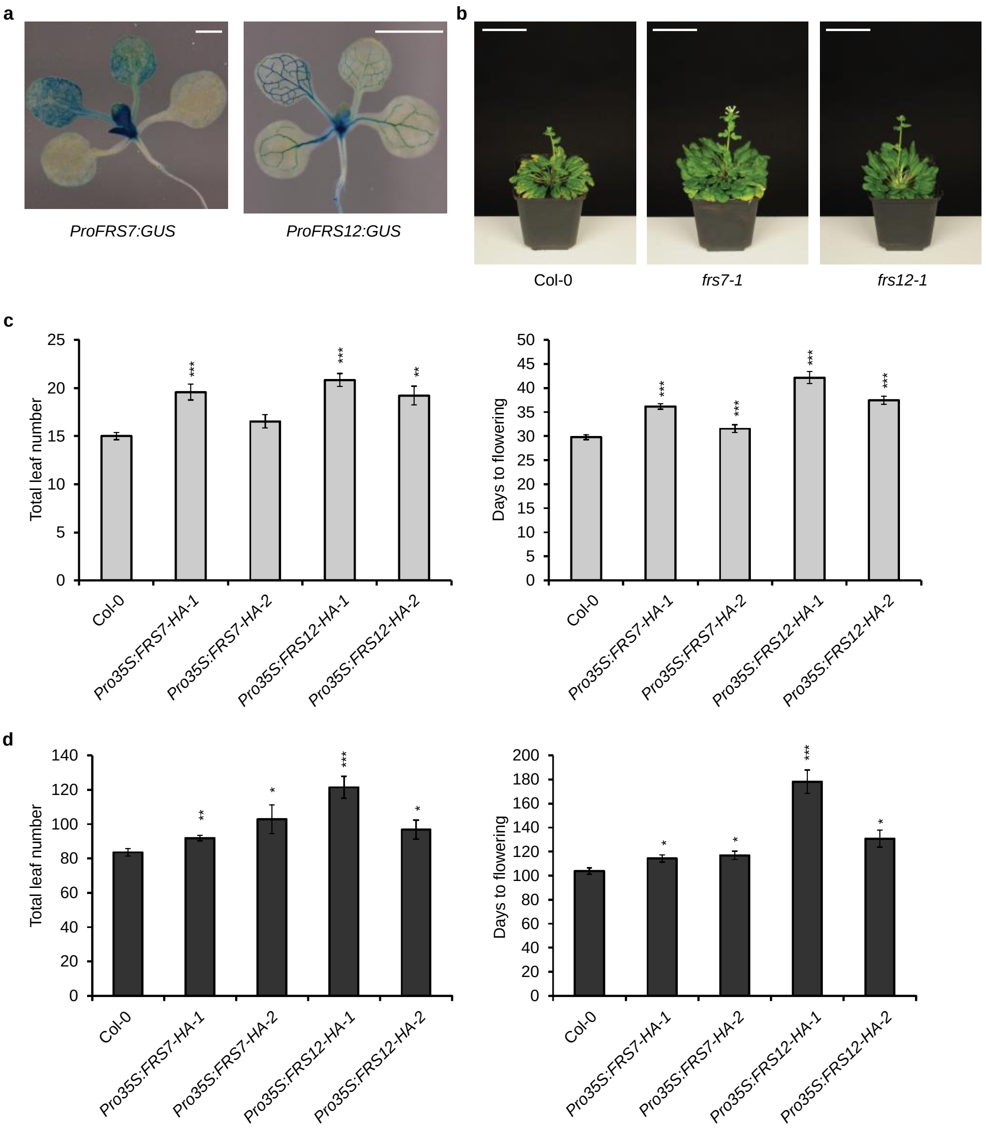

Supplementary Figure 4. FRS7 and FRS12 are flowering time modulators. a, β-Glucuronidase (GUS) histochemical analysis of the spatial expression of *FRS7* and *FRS12* in 14-day-old seedlings. Scale bars: 1 mm. **b**, Representative photographs comparing a SD-grown Col-0 wt flowering plant to the *frs7-1* and *frs12-1* single mutant lines. Scale bars: 5 cm. **c**, Flowering time measurements as total leaf number (left panel) and days to flower (right panel) of LD-grown Col-0 wt *Arabidopsis* plants compared to gain-of-function lines of *FRS7*, *FRS12*. **d**, Flowering time measurements as total leaf number (left panel) and days to flower (right panel) of SD-grown Col-0 wt *Arabidopsis* plants compared to gain-of-function lines of *FRS7*, *FRS12*.Values represent the average of 12 biological replicates ± SEM; *P<0.05, **P<0.01, ***P<0.001, t-test.

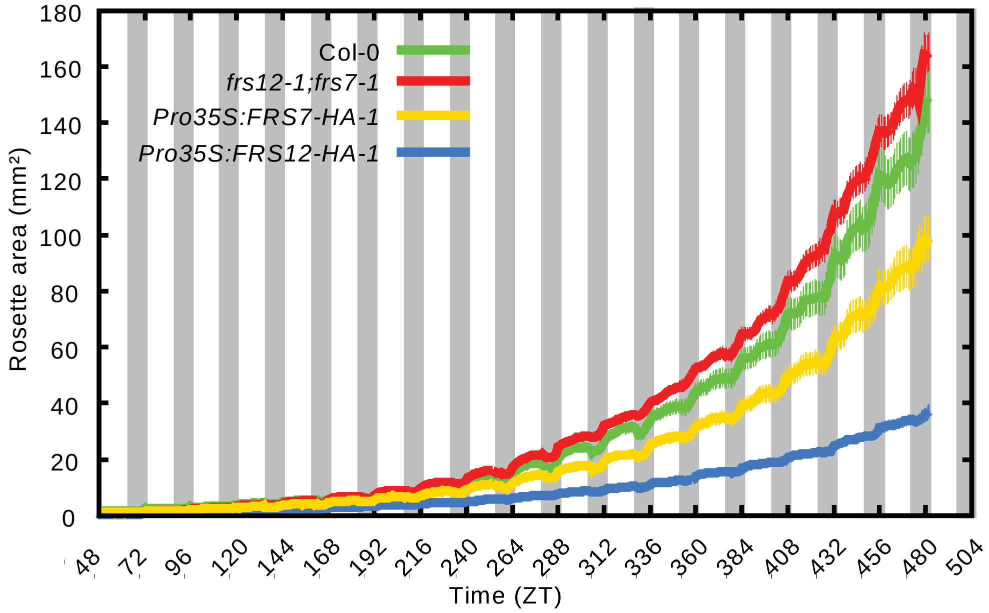

Supplementary Figure 5. FRS7 and FRS12 modulate leaf-rosette growth. Rosette leaf growth dynamics of LD-growing *Arabidopsis*. Col-0 wt seedlings were grown in parallel to the *frs7-1;frs12-1* double mutant and *Pro35S:FRS7-HA-1* and *Pro35S:FRS12-HA-1* overexpressing lines. Gray bands represent night periods. Values represent the average of 25 biological replicates ± SEM.

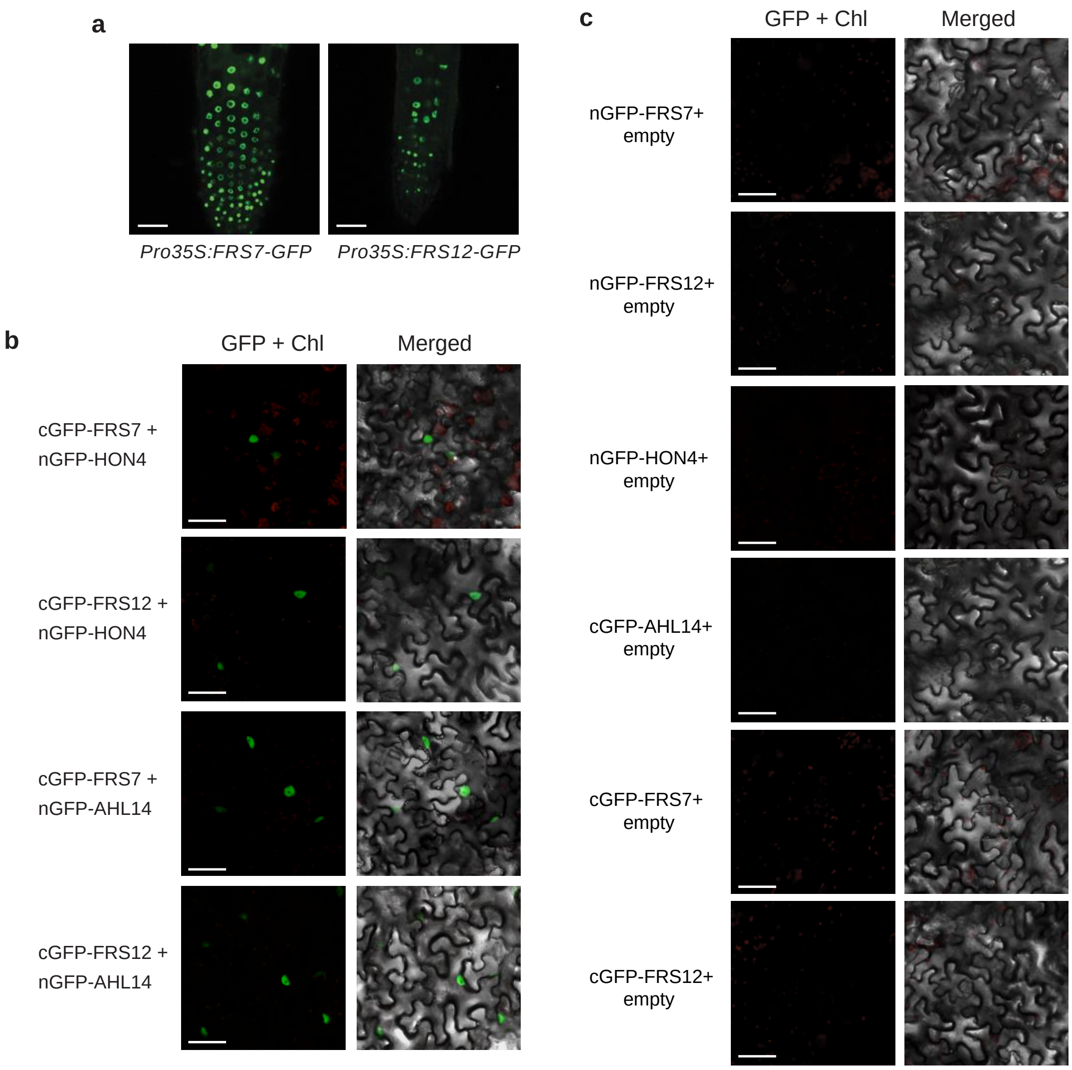

Supplementary Figure 6. FRS7 and FRS12 are nuclear-localized proteins interacting with HON4 and AHL14. a, Laser confocal microscope images of *Arabidopsis* primary root cells constitutively expressing *FRS7-GFP* (left) and *FRS12-GFP* (right). Scale bars: 30 μm. **b**, BiFC analysis in *N. benthamiana* leaves of FRS7-HON4, FRS12-HON4, FRS7-AHL14 and FRS12-AHL14 nuclear interactions. **c**, Negative BiFC controls of single expressed nGFP-FRS7, nGFP-FRS12, nGFP-HON4, nGFP-AHL14, cGFP-FRS7 and cGFP-FRS12. Scale bars: 50 μm.

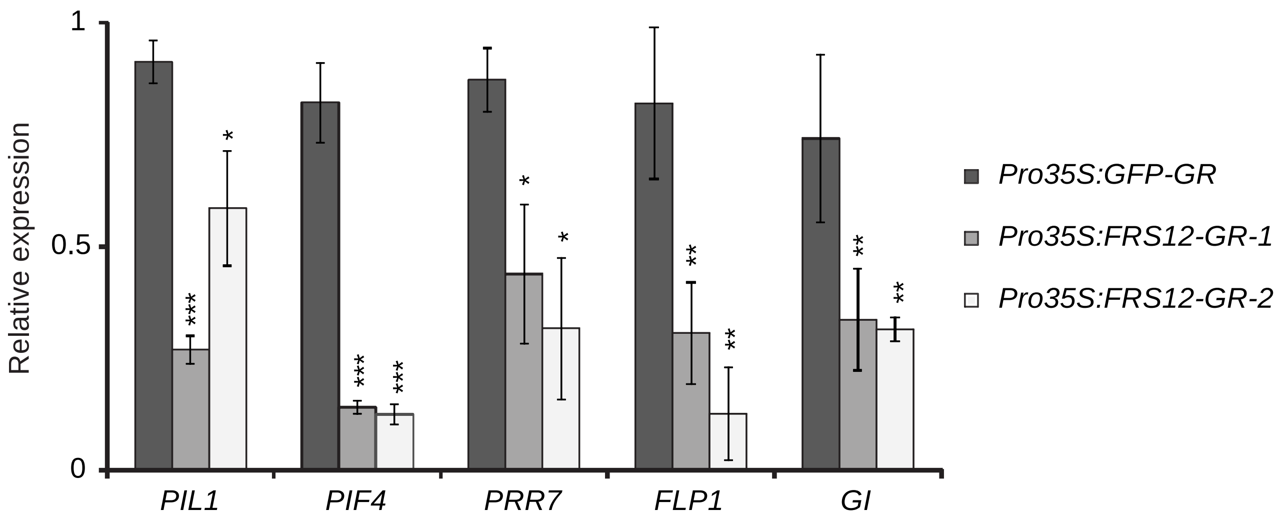

Supplementary Figure 7. Induced overexpression of *FRS12* represses light and clock-related genes. *Pro35S:FRS12-GR-1, Pro35S:FRS12-GR-2* and *Pro35S:GFP-GR* (control) lines were grown for one week under long-days, then treated with 5 µM of DEX and harvested at four hours after treatment (ZT21). Expression values of *PIL1, PIF4, PRR7, FLP1* and *GI* were evaluated and normalized to the expression of *UBC* (AT5G25760) and *PP2A* (At1g13320) as internal expression controls. Values represent the average expression of 3 biological replicates ± SEM; *P<0.05, **P<0.01, t-test.

a

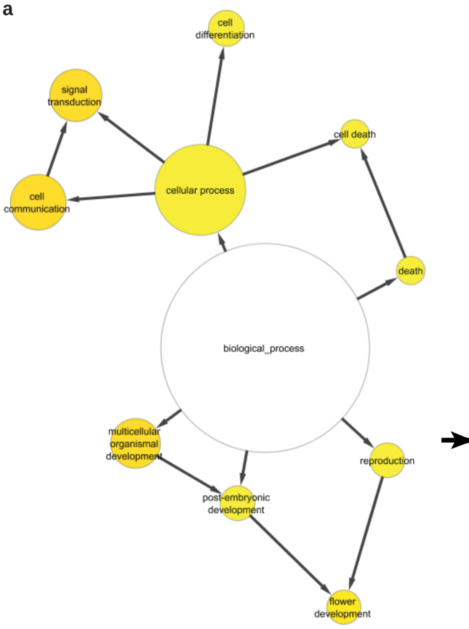

b

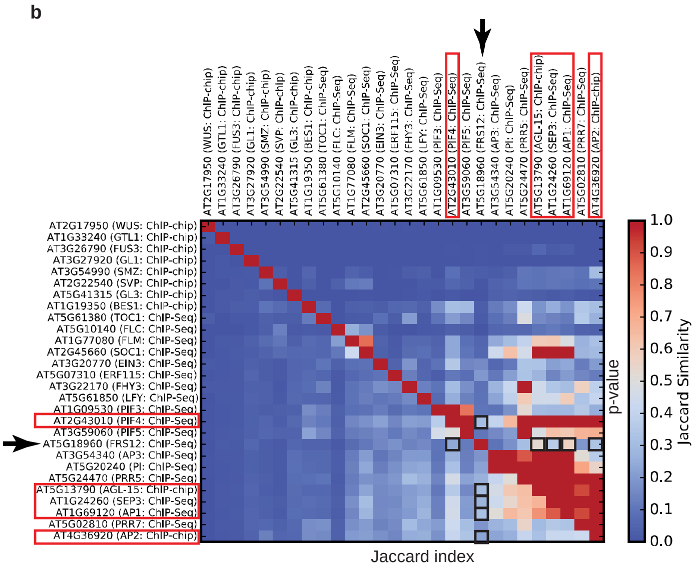

c

TF1				TF2							
Locus	Name	Experiment	Bound loci	Locus	Name	Experiment	Bound loci	Gene intersection	P value	Correction	Transf
AT3G26790	FUS3	ChIP-chip	108	AT5G18960	FRS12	ChIP-Seq	2,743	23	9.91E-05	4.03E-02	1.40
AT3G27920	GL1	ChIP-chip	671	AT5G18960	FRS12	ChIP-Seq	2,743	91	9.39E-05	3.81E-02	1.42
AT3G54990	SMZ	ChIP-chip	140	AT5G18960	FRS12	ChIP-Seq	2,743	42	2.14E-12	8.70E-10	9.06
AT2G22540	SVP	ChIP-chip	59	AT5G18960	FRS12	ChIP-Seq	2,743	16	5.52E-05	2.24E-02	1.65
AT1G19350	BES1	ChIP-chip	299	AT5G18960	FRS12	ChIP-Seq	2,743	67	4.01E-12	1.63E-09	8.79
AT5G61380	TOC1	ChIP-Seq	333	AT5G18960	FRS12	ChIP-Seq	2,743	67	5.73E-10	2.33E-07	6.63
AT1G77080	FLM	ChIP-Seq	562	AT5G18960	FRS12	ChIP-Seq	2,743	118	5.97E-18	2.42E-15	14.62
AT2G45660	SOC1	ChIP-Seq	1,026	AT5G18960	FRS12	ChIP-Seq	2,743	192	2.92E-22	1.18E-19	18.93
AT3G20770	EIN3	ChIP-Seq	1,062	AT5G18960	FRS12	ChIP-Seq	2,743	262	1.94E-52	7.87E-50	49.10
AT5G07310	ERF115	ChIP-Seq	1,408	AT5G18960	FRS12	ChIP-Seq	2,743	335	2.43E-63	9.85E-61	60.01
AT3G22170	FHY3	ChIP-Seq	1,469	AT5G18960	FRS12	ChIP-Seq	2,743	275	1.02E-31	4.15E-29	28.38
AT5G61850	LFY	ChIP-Seq	1,760	AT5G18960	FRS12	ChIP-Seq	2,743	313	7.14E-32	2.90E-29	28.54
AT1G09530	PIF3	ChIP-Seq	639	AT5G18960	FRS12	ChIP-Seq	2,743	164	3.96E-35	1.61E-32	31.79
**AT2G43010**	**PIF4**	**ChIP-Seq**	**2,200**	**AT5G18960**	**FRS12**	**ChIP-Seq**	**2,743**	**519**	**2.50E-99**	**1.01E-96**	**95.99**
AT3G59060	PIF5	ChIP-Seq	808	AT5G18960	FRS12	ChIP-Seq	2,743	192	5.21E-36	2.12E-33	32.67
AT5G18960	FRS12	ChIP-Seq	2,743	AT3G54340	AP3	ChIP-Seq	3,720	551	2.05E-33	8.31E-31	30.08
AT5G18960	FRS12	ChIP-Seq	2,743	AT5G20240	PI	ChIP-Seq	4,202	681	5.24E-57	2.13E-54	53.67
AT5G18960	FRS12	ChIP-Seq	2,743	AT5G24470	PRR5	ChIP-Seq	5,930	992	4.73E-99	1.92E-96	95.72
**AT5G18960**	**FRS12**	**ChIP-Seq**	**2,743**	**AT5G13790**	**AGL-15**	**ChIP-chip**	**6,790**	**1,234**	**1.48E-164**	**6.02E-162**	**161.22**
**AT5G18960**	**FRS12**	**ChIP-Seq**	**2,743**	**AT1G24260**	**SEP3**	**ChIP-Seq**	**4,321**	**837**	**1.30E-115**	**5.27E-113**	**112.23**
**AT5G18960**	**FRS12**	**ChIP-Seq**	**2,743**	**AT1G69120**	**AP1**	**ChIP-Seq**	**4,615**	**991**	**2.09E-176**	**8.47E-174**	**173.07**
AT5G18960	FRS12	ChIP-Seq	2,743	AT5G02810	PRR7	ChIP-Seq	1,760	383	1.15E-61	4.69E-59	58.33
**AT5G18960**	**FRS12**	**ChIP-Seq**	**2,743**	**AT4G36920**	**AP2**	**ChIP-chip**	**1,532**	**425**	**1.10E-104**	**4.47E-102**	**101.35**

d

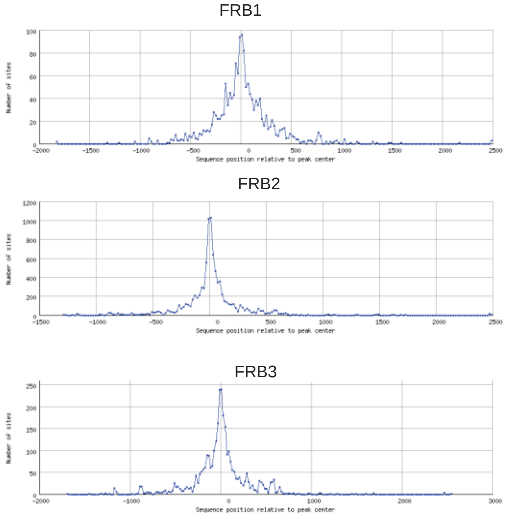

Supplementary Figure 8. FRS12 binds *in vivo* to genes related to flowering time and diurnal growth pathways. **a**, GOslim enrichment diagram of the FRS12-HBH TChAP-Seq-bound genes. Node sizes are proportional to the enriched gene number and the yellow color intensity is proportional to the *P*-value significance. **b**, Transcription factor co-binding matrix for common potential target genes created by average-linkage hierarchical clustering based on the Jaccard index. The lower left half displays the Jaccard index, while the upper right half displays hypergeometric *P*-values of overlap between the two sets of bound genes, corrected using the Bonferroni method. Black arrows highlight FRS12 and red rectangles highlight the five TFs presenting the target genes most significantly co-bound to FRS12. **c,** Statistical results highlighting the TFs that share potential target genes with FRS12. Results describe common potential target genes and average-linkage hierarchical clustering analysis based on the Jaccard index. Bold lines highlight the top 5 transcription factors presenting the most significant co-binding values to FRS12. **d**, Distribution of the FRB1, FRB2 and FRB3 motifs in relation to the peak summits.

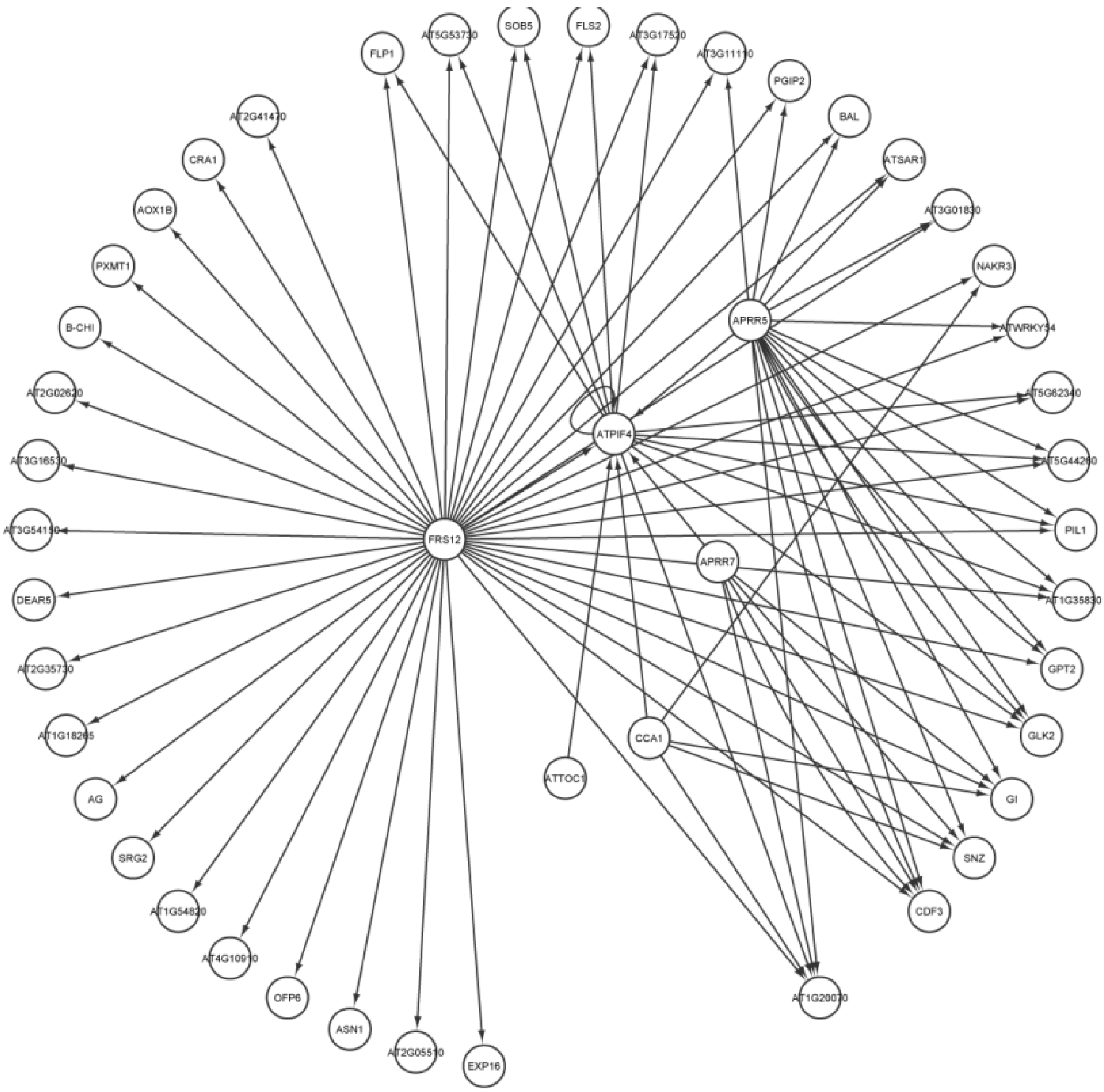

Supplementary Figure 9. Gene regulatory network of FRS12, PIF4 and circadian clock components. Circles inside the network and surrounding circles represent transcription factors and targets of (genes bound and regulated) FRS12, respectively. An arrow indicates a regulatory interaction based on ChIP-Seq.

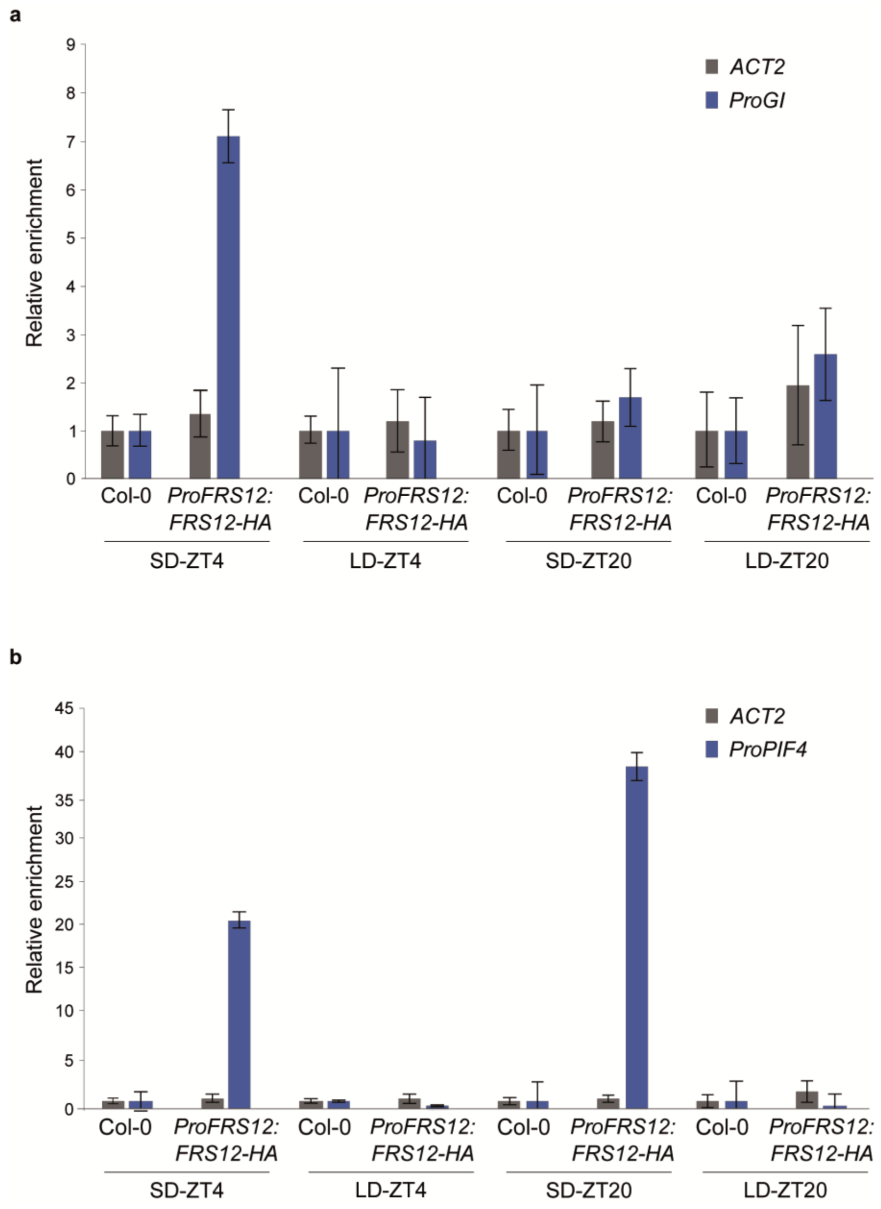

Supplementary Figure 10. FRS12 binds promoters of genes responsible for diurnal growth and flowering in a photoperiodic-dependent manner. ChIP-qPCR assay of selected fragments in the *GI* (**a**) and *PIF4* (**b**) promoters. The transgenic *Arabidopsis* line *ProFRS12:FRS12-HA* was grown for 10 days in SD and LD and harvested at ZT4 and ZT20 for analysis. Enrichment values were normalized to respective inputs and represented relative to Col-0 wt plants (background control). Values represent the mean of 3 biological replicates ± SEM.

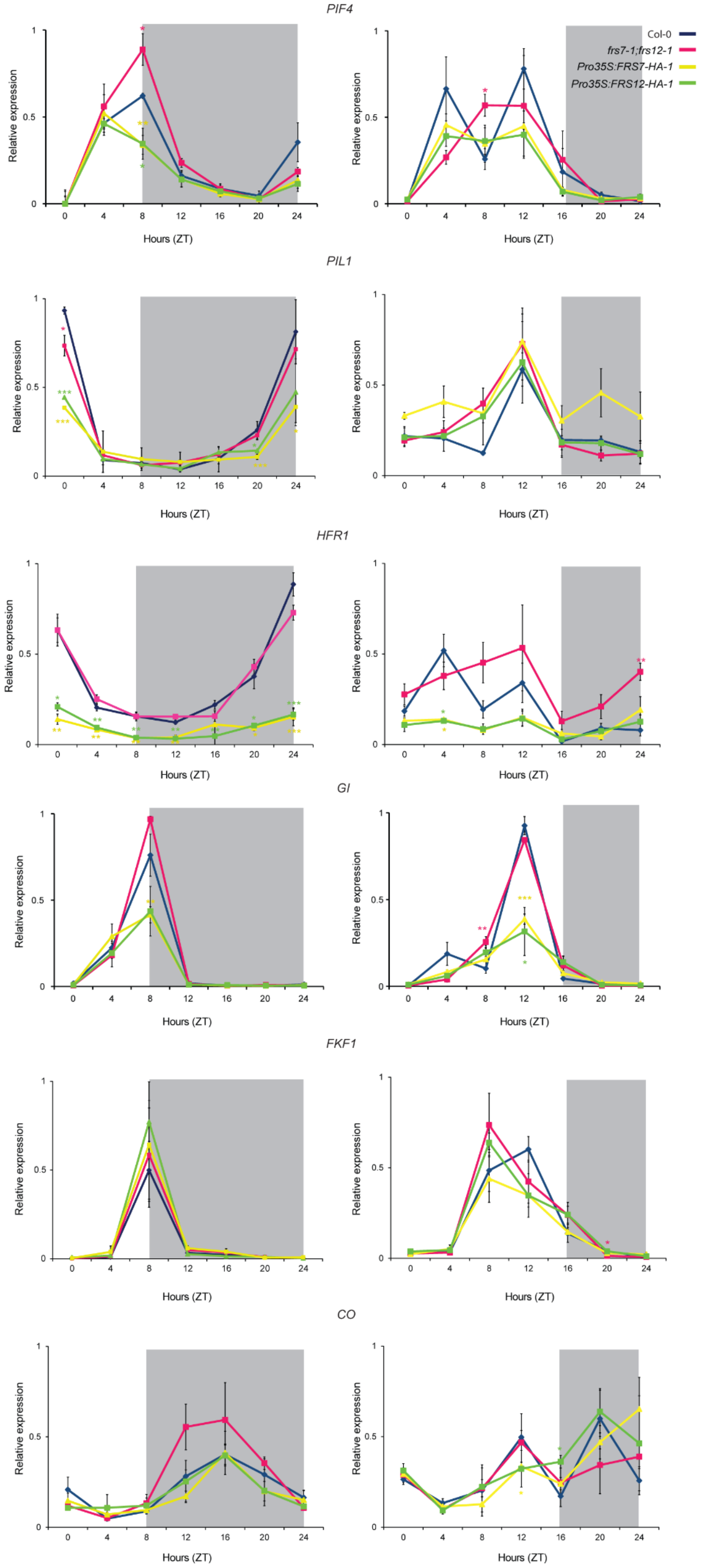
PIF4
PIL1
HFR1
GI
FKF1
CO
Col-0
frs7-1;frs12-1
Pro35S:FRS7-HA-1
Pro35S:FRS12-HA-1
Relative expression
Hours (ZT)

Supplementary Figure 11. Effect of ectopic expression of *FRS7* and *FRS12* on target genes. Diurnal oscillations of *PIF4*, *GI*, *PIL1*, *HFR1*, *FKF1* and *CO* transcript levels in Col-0 wt seedlings compared to the double *frs7-1;frs12-1* mutant, and *Pro35S:FRS7-HA-1* and *Pro35S:FRS12-HA-1* overexpressing lines grown under SD (left panels) or LD (right panels). Gray rectangles represent the dark period. Values represent the average expression of 3 biological replicates ± SEM; **P*<0.05, ***P*<0.01, t-test. "1" represents the highest level of expression for a particular gene.

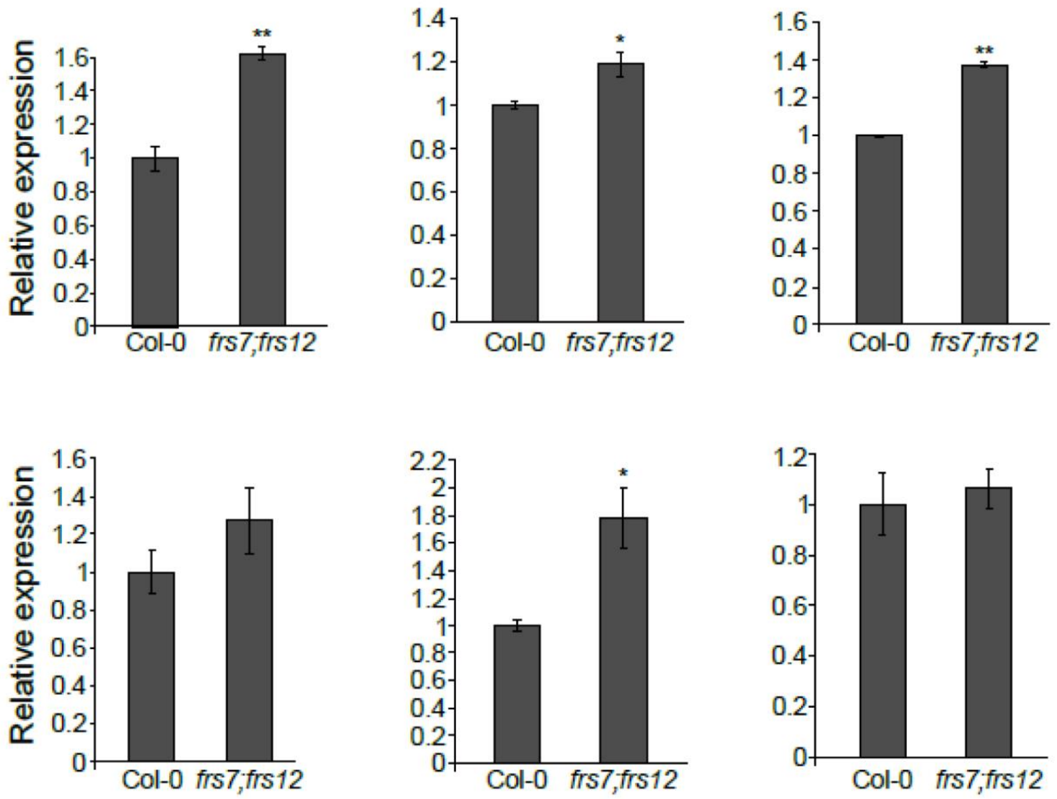

Supplementary Figure 12. Effects of loss-of-function of *FRS7* and *FRS12* on *PIF4* expression. *PIF4* transcript levels in the double *frs7-1;frs12-1* mutant compared to Col-0 wt seedlings (set at 1) grown under SD and harvested at ZT8 in 6 independent experiments. Values represent the average expression of 2 to 4 biological replicates ± SEM; **P*<0.05, ***P*<0.01, t-test.

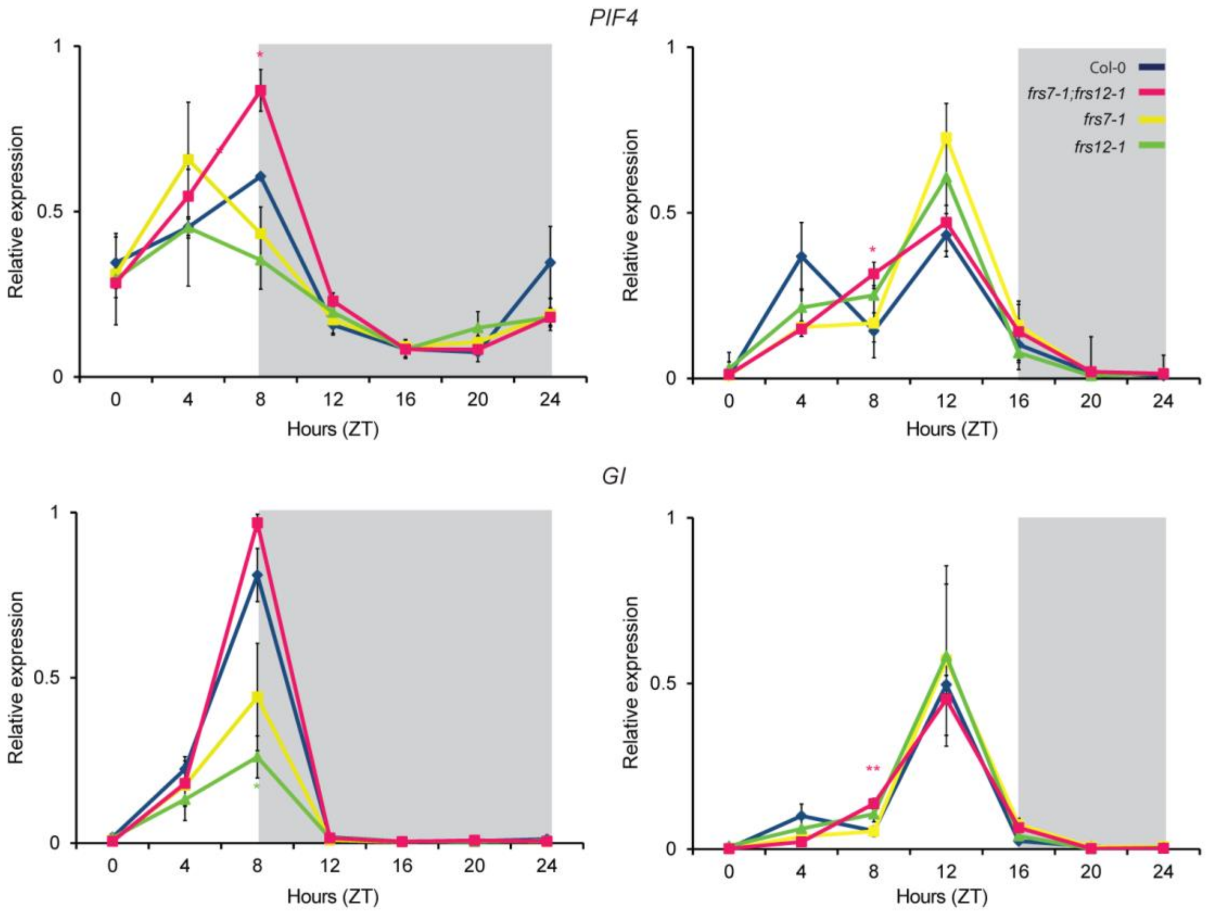

Supplementary Figure 13. Cooperative functions of FRS7 and FRS12 to repress diurnal growth and photoperiodic flowering pathways. Diurnal oscillations of *PIF4* and *GI* transcript levels in Col-0 wt seedlings compared to the single *frs7-1* and *frs12-1* and the double *frs7-1;frs12-1* mutants grown under SD (left panels) or LD (right panels). Gray rectangles represent the dark period. Values represent the average expression of 3 biological replicates ± SEM; **P*<0.05, ***P*<0.01, t-test. "1" represents the highest level of expression for a particular gene.

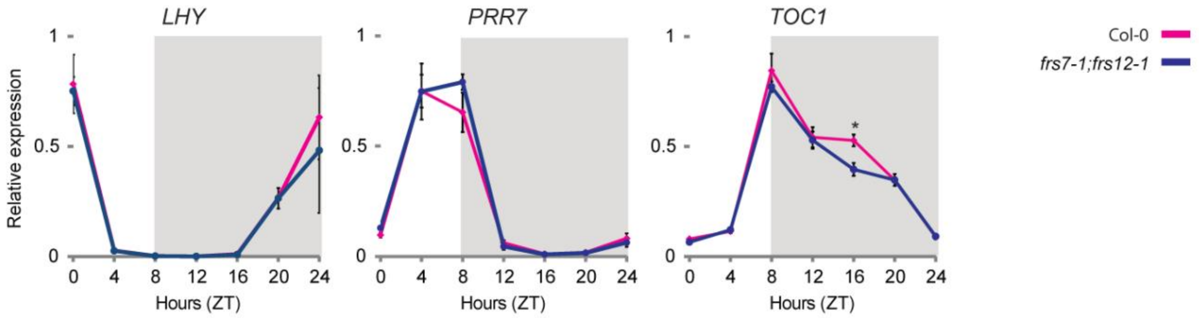

Supplementary Figure 14. FRS7-FRS12 do not affect the circadian clock functioning. Diurnal oscillations of transcript levels of circadian clock genes in Col-0 wt seedlings compared to the double *frs7-1;frs12-1* mutant. Gray rectangles represent the dark period. Values represent the average expression of 3 biological replicates ± SEM; **P*<0.05, ***P*<0.01, t-test. "1" represents the highest level of expression for a particular gene.

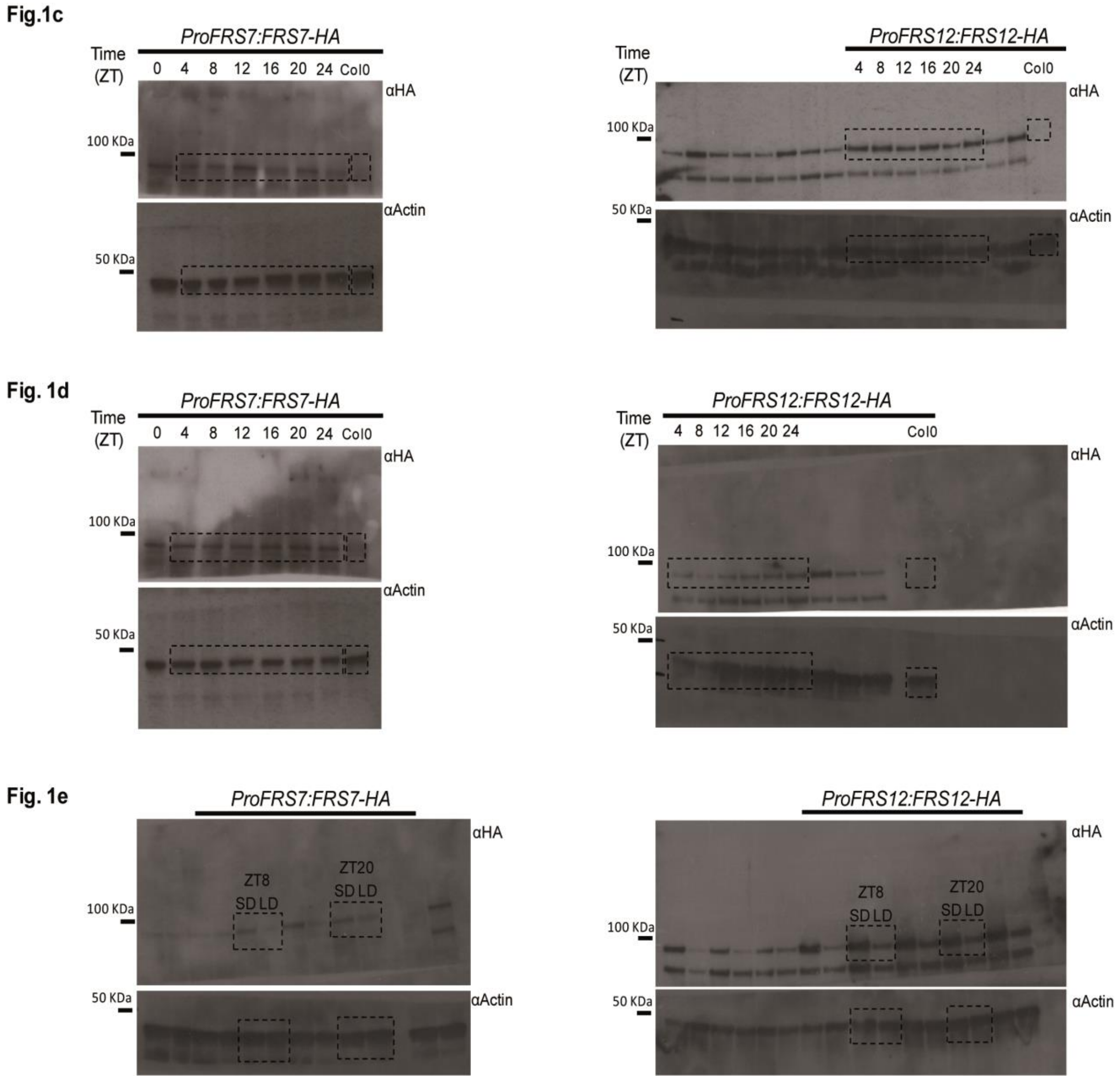

Supplementary Figure 15. Original images of cropped immunoblot figures shown in Fig 1.

Supplementary Table 1. Statistical results of leaf series measurements.

a

Individual leaf		*Pro35S*:FRS7-HA-1	*Pro35S*:FRS12-HA-1	*frs7-1;frs12-1*
	Cot	**3.29169E-05**	**0.001190801**	**0.034783184**
	1	**2.06482E-05**	**0.029987748**	**0.032224747**
	2	**1.70106E-05**	**0.023917684**	**0.03610006**
	3	**1.16242E-05**	**0.016156871**	**0.045844491**
	4	**7.2757E-05**	**0.014715746**	**0.049654893**
	5	**7.88528E-06**	**0.012938938**	0.050967207
	6	**9.81655E-06**	**0.013072534**	0.050349739
	7	**1.22625E-05**	**0.013686145**	**0.04846985**

**Total area**	Average (mm)^2	Ratio relative to Col-0	*P*-value
**Col-0**	1719.203		
***Pro35S*:FRS7-HA-1**	907.929	0.528110409	**0.000103789**
***Pro35S*:FRS12-HA-1**	1259.969	0.732879712	**0.010014974**
frs7-1;frs12-1	2551.396	1.484057438	**0.013542658**

b

Individual leaf		*Pro35S*:FRS7-HA-1	*Pro35S*:FRS12-HA-1	*frs7-1;frs12-1*
	Cot	**0.018858458**	**0.016404643**	0.435493988
	1	0.08458914	**0.015431255**	0.41668861
	2	0.081786723	**0.014564198**	0.422435475
	3	0.069092989	**0.011941888**	0.477118875
	4	0.053956106	**0.008552939**	0.549997227
	5	**0.039557143**	**0.0060756**	0.583697343
	6	**0.027829936**	**0.005713693**	0.588139787
	7	**0.026259078**	**0.005446892**	0.583305215
	8	**0.031047416**	**0.007962883**	0.549917485
	9	**0.032586113**	**0.009479576**	0.558391896
	10	**0.036380884**	**0.011764264**	0.473699644

**Total area**	Average (mm)^2	Ratio relative to Col-0	*P*-value
**Col-0**	1719.025		
***Pro35S*:FRS7-HA-1**	1254.442	0.729740405	0.085297471
***Pro35S*:FRS12-HA-1**	1207.806	0.702611073	**0.023221389**
frs7-1;frs12-1	1749.733	1.017863615	0.881934952

P-values (t-test) showing differences in individual leaf areas and in total leaf rosette area between *FRS7* and *FRS12* altered lines and Col-0 wt grown under LD (**a**) or SD (**b**) conditions (n=8 plants/genotype).

Supplementary Table 2. Unique peptides identified in TAP-MS experiments.

AGI code	Name	Unique Peptide Sequence	In TAP experiments
**AT3G04590**	AHL14	ELAAVTGGTVSTNSGSSK	3, 9
**AT3G04590**	AHL14	IGHESSENGDYEQQIPD	3, 9
**AT2G45850**	AHL9	TGNLSVSLASPDGR	9, 10
**AT2G45850**	AHL9	VIAFSQQGPR	9, 10
**AT5G18960**	FRS12	ALMVWSLR	5, 6, 9, 10
**AT5G18960**	FRS12	AVTGTEPYAGLEFGSANEACQFYQAYAEVVGFRVR	3
**AT5G18960**	FRS12	DDVWLR	9
**AT5G18960**	FRS12	DMESGVSAQDLK	10
**AT5G18960**	FRS12	EFYNAYAAR	10
**AT5G18960**	FRS12	EHNHELGGEGSVEETTPR	5, 6, 9, 10
**AT5G18960**	FRS12	EHNHELGGEGSVEETTPRPSR	5, 6, 9
**AT5G18960**	FRS12	ENLIPFPSEFK	9
**AT5G18960**	FRS12	FKGGGGEGEVSDDHHQTQQAK	9
**AT5G18960**	FRS12	IFQNELVQSYNYLCLK	9
**AT5G18960**	FRS12	LGVTVNPHRPK	6, 9
**AT5G18960**	FRS12	LYTLTVFR	5, 9
**AT5G18960**	FRS12	QPVLLGCAMVADESK	2, 3, 9, 10
**AT5G18960**	FRS12	YEQALEQR	3, 8, 9, 10
**AT5G18960**	FRS12	YSAWQIR	1, 2, 3, 4, 5, 6, 7, 9, 10
**AT3G06250**	FRS7	DVESGVTSQDLK	4, 7, 8, 9, 10
**AT3G06250**	FRS7	FSAWQIR	9
**AT3G18035**	HON4	DGVTSENQAVVQAIK	9, 10
**AT3G18035**	HON4	IGGVISR	7
**AT3G18035**	HON4	IGTSVTTGTQDSGELK	10
**AT3G18035**	HON4	SEILHSSNNDPMASGSASQPLK	9
**AT3G18035**	HON4	SVSSTASVYPYVANGAR	7, 9, 10

Supplementary Table 3. Genes physically bound and transcriptionally regulated by FRS12.

Locus	Short description	RNA_Seq Fold Change
**AT4G31380**	FPF1-like protein 1 (FLP1)	-23,33
**AT2G43010**	Phytochrome interacting factor 4 (PIF4)	-9,64
**AT1G22770**	GIGANTEA (GI)	-5,26
**AT3G55500**	Expansin A16 (EXPA16)	-4,46
**AT2G05510**	Glycine-rich protein family	-4,13
**AT2G46970**	Phytochrome interacting factor 3-like 1 (PIL1)	-4,08
**AT3G11110**	RING/U-box superfamily protein	-3,56
**AT3G47340**	Glutamine-dependent asparagine synthase 1 (ASN1)	-3,53
**AT3G53530**	Chloroplast-targeted copper chaperone protein	-2,74
**AT1G35830**	VQ motif-containing protein	-2,57
**AT5G53730**	Late embryogenesis abundant (LEA) hydroxyproline-rich glycoprotein family	-2,56
**AT5G06870**	Polygalacturonase inhibiting protein 2 (PGIP2)	-2,47
**AT5G44260**	Zinc finger C-x8-C-x5-C-x3-H type family protein	-2,46
**AT3G52525**	Ovate family protein 6 (OFP6)	-2,37
**AT5G08150**	SUPPRESSOR OF PHYTOCHROME B 5 (SOB5)	-2,32
**AT5G44190**	GOLDEN2-like 2 (GLK2)	-2,30
**AT5G46330**	FLAGELLIN-SENSITIVE 2 (FLS2)	-2,27
**AT1G20070**	unknown protein	-2,21
**AT4G10910**	unknown protein	-2,20
**AT1G54820**	Protein kinase superfamily protein	-2,18
**AT3G60140**	DARK INDUCIBLE 2 (DIN2)	-2,12
**AT3G47500**	cycling DOF factor 3 (CDF3)	-2,07
**AT4G18960**	AGAMOUS (AG)	-2,07
**AT2G39250**	SCHNARCHZAPFEN (SNZ)	-2,01
**AT1G18265**	Protein of unknown function, DUF593	2,02
**AT2G35730**	Heavy metal transport/detoxification superfamily protein	2,14
**AT5G62340**	Plant invertase/pectin methylesterase inhibitor superfamily protein	2,15
**AT1G09180**	secretion-associated RAS super family 1 (SARA1A)	2,15
**AT3G17520**	Late embryogenesis abundant protein (LEA) family protein	2,19
**AT4G06746**	related to AP2 9 (RAP2.9)	2,22
**AT2G40750**	Member of WRKY Transcription Factor; Group III (WRKY54)	2,25
**AT3G54150**	S-adenosyl-L-methionine-dependent methyltransferases superfamily protein	2,33
**AT3G01830**	Calcium-binding EF-hand family protein	2,39
**AT2G45760**	BON association protein 2 (BAP2)	2,41
**AT3G16530**	Legume lectin family protein	2,41
**AT2G02620**	Cysteine/Histidine-rich C1 domain family protein	2,83
**AT3G12500**	basic chitinase (HCHIB)	3,06
**AT1G66700**	PXMT1	3,56
**AT3G22360**	alternative oxidase 1B (AOX1B)	3,88
**AT1G61800**	glucose-6-phosphate/phosphate translocator 2 (GPT2)	6,00
**AT5G44120**	CRUCIFERINA (CRA1)	11,31
**AT2G41470**	unknown protein	13,27

Supplementary Table 4. Primers used in this study.

Name	Sequence 5' 3'	Type	Target sequence
**1209**	ATTGACATCCAATTCGACAGC	FW	SALK_030182.42.45.x (*frs12-1* genotyping)
**1210**	GTTCTTGTGTTCGTTGGCTTC	RV	SALK_030182.42.45.x (*frs12-1* genotyping)
**669**	ATTTTGCCGATTTCGGAAC	RV	LBb1.3 SALK T-DNA primer
**1618**	TGAAACAACCATGAGAAAGCC	FW	LP_FLAG_196C09 (*frs7-1* genotyping)
**1619**	CAACTCTTATGCTACGCGGAC	RV	LP_FLAG_196C09 (*frs7-1* genotyping)
**1621**	CGTGTGCCAGGTGCCCACGGAATAGT	RV	FST_LB4 T-DNA primer
**1124**	GGGGACAAGTTTGTACAAAAAAGCAGGCTCCATGGAGAGTGTAGATACTGA G	FW	attB1-FRS12
**1125**	GGGGACCACTTTGTACAAGAAAGCTGGGTCTCMTCTCTGCCAACAAAGTTTC	RV	attB2-FRS12
**1555**	AAGGGATCCGGCGTATCACTAACTCAAAAAACT	FW	FRS12 promoter (-0-585bp) +BamHI
**1556**	GGTCTCGAGTCTCGTCGAAGCGACCACCAAAGA	RV	FRS12 promoter (0-582bp) + XhoI
**1557**	AAGGGATCCAGACCATGTCCTTTGGAAAG	FW	FRS7 promoter (-800-0) +BamHI
**1558**	GTGCTCGAGGTTGTTCCCACAATTTAAAC	RV	FRS7 promoter (-800-0)+ XhoI
**2418**	GGGGACAAGTTTGTACAAAAAAGCAGGCTCCTCATGCTCATCTAAGGATGAC A	FW	attB1 -3000bp ProPIF4
**2748**	GGGGACCACTTTGTACAAGAAAGCTGGGTCCCTCTCCAAATGAAATGAACTT CCTTATATAGAGGAAGGGTCTTGCgtcagatctctggagacatt	RV	attB2_Min35S_ProPIF4
**1816**	TCAGATGCAGCCGATGGAGATG	FW	qPCR primer PIF4
**1817**	CGACGGTTGTTGACTTTGCTGTC	RV	qPCR primer PIF4
**1818**	TCGTGGTGCCTTCGTGTGTTTC	FW	qPCR primer PIL1
**1819**	CGGACGCAGACTTTGGGAATTG	RV	qPCR primer PIL1
**1806**	ACTCTACACGGTTTCCTTATCCT	FW	qPCR primer FLP1
**1807**	AATACCCACACACCAGACATTG	RV	qPCR primer FLP1
**1808**	GTGTTGACTGTATGTGTTAG	FW	qPCR primer GI
**1809**	GTTAGCAGTTTGATTGTTAGA	RV	qPCR primer GI
**1810**	ATCATAATAATCATGCCTCCTAT	FW	qPCR primer PRR7
**1811**	TTGTTGTTACCTTCAATCGT	RV	qPCR primer PRR7
**2224**	TCATCTCCGATATCTCTTTAACTAACA	FW	qPCR primer HFR1
**2225**	TAGACGATCTTCATCACTTCTTGC	RV	qPCR primer HFR1
**3067**	GTTGTACCGCCTCCAAGACT	FW	qPCR primer FKF1
**3068**	AGATGATGACCCTACCACACG	RV	qPCR primer FKF1
**1753**	CAATGGTTCCATTAACCATAACGCATA	FW	qPCR primer CO
**1754**	CTTATCTCTGCATATGCCTTCCTCGAA	RV	qPCR primer CO
**2686**	GAGCTTGGCAACGAATTGAAGAAC	FW	qPCR primer LHY
**2687**	AAAGCTTGGCAAACAGGGATGC	RV	qPCR primer LHY
**2690**	TTAGGTCCACCAACCCACAGAGAG	FW	qPCR primer TOC1
**2691**	AGGAGCAGTAGCAACAGACCACTC	RV	qPCR primer TOC1
**1082**	CTGCGACTCAGGGAATCTTCTAA	FW	qPCR primer UBC
**1083**	TTGTGCCATTGAATTGAACCC	RV	qPCR primer UBC
**1084**	TAACGTGGCCAAAATGATGC	FW	qPCR primer PP2A
**1085**	GTTCTCCACAACCGCTTGGT	RV	qPCR primer PP2A

**2746**	TTGACTACGAGCAGGAGATGG	FW	qPCR primer ACT2
**2747**	ACAAACGAGGGCTGGAACAAG	RV	qPCR primer ACT2
**2732**	GAGCATTGAACTCGGATAA	FW	ProPIF4_region2_ChIP-qPCR
**2733**	GATTTGAGGGTGTTTTTGTC	RV	ProPIF4_region2_ChIP-qPCR
**2777**	GACCAAAACAAATCCTCCA	FW	ProPil1_FRB23-ChIP-qPCR
**2778**	GATTCGGACTTCACACTT	RV	ProPil1_FRB23-ChIP-qPCR
**2720**	GTAGAGACAAGTGGTAAGA	FW	ProGI_FRB1_ChIP-qPCR
**2721**	TTGTAGATAAACGGGCAG	RV	ProGI_FRB1_ChIP-qPCR
	CCATGGTTAATTAAGACGTCGAACCGCAACGTTGAAGGAGC	Fw	NptII-F
	AAACACTGATAGTTTAAACGATCTAGTAACATAGATGACACCGCGC	Rv	NptII-R
	GGGGACAAGTTTGTACAAAAAAGCAGGCTTACTTTTTTTCTTCTTCTTCGTTC ATACAG	FW	attB1_AtU6gRNA
	GGGGACAACTTTTGTATACAAAGTTGTGTCTAGAAAAAAAGCACCGACTCGG	RV	attB5r_AtU6gRNA
	GGGGACAACTTTGTATACAAAAGTTGTACTTTTTTTCTTCTTCTTCGTTCATAC AG	FW	attB5_AtU6gRNA
	GGGGACCACTTTGTACAAGAAAGCTGGGTTCTAGAAAAAAAGCACCGACTCG G	RV	attB2_AtU6gRNA
	AGTCTTGCGACTGAGCCTTTCGTTTTATTTGATGCC	FW	noBbsI_F
	CTCAGTCGCAAGACTGGGCCTTTCGTTTTATCTG	RV	noBbsI_R
**2644**	**ATTG**TAGGAGCTGGAGCTCTCGA	FW	FRS12 gRNA 14
**2645**	**AAAC**TCGAGAGCTCCAGCTCCTA	RV	FRS12 gRNA 14
**2646**	**ATTG**AGCACAACCATGACCTTGG	FW	FRS7 gRNA 196
**2647**	**AAAC**CCAAGGTCATGGTTGTGCT	RV	FRS7 gRNA 196
**2970**	TGGTACGGTTCGTCTAGGAG	FW	FRS12-14 TIDE
**2971**	ACCAGCGTCATTCTTCTTCC	RV	FRS12-14 TIDE
**3115**	TGTTTCTGTGTCCAAGAATGTG	FW	FRS7 TIDE
**3116**	AACCCTGCATACGGTTCAGT	RV	FRS7 TIDE
**3075**	TCCCTCATCAGATCCACCTC	FW	Cas9 genotyping
**3076**	CTGAAACCTGAGCCTTCTGG	RV	Cas9 genotyping
**2968**	CGGACGGGTTTTAAGGTTAG	FW	RFLP primer FRS7 3-11 BsaJI
**3117**	TGCCCATCACTATCTTCAGC	RV	RFLP primer FRS7 3-11 BsaJI
**3118**	AACCATGAGCTTGGAGGTGA	FW	RFLP primer FRS12 3-11 Hpy188III
**3119**	GATGTGATCGAACCGTCAAC	RV	RFLP primer FRS12 3-11 Hpy188III

Supplementary Table 5. Plasmids used in this study.

Code	Vector	Insert
**Z2066**	pENTR223.1-Sfi	FRS7
**Z2399**	pDONR207	FRS7 (NO STOP)
**Z2593**	pFAST-R05	FRS7(NO STOP)
**Z3174**	pDONR207	FRS7 (STOP)
**Z3203**	pENL4R1	ProFRS7 (0-800bp)
**Z3214**	pK8m34GW-FAST	35S:FRS7-3HA
**Z3222**	pmK7S*NFm14GW	ProFRS7 (0-800bp)
**Z3437**	pK8m34-Fast	ProFRS7:FRS7-3HA
**Z4073**	pm42GW7	ProFRS7:LUC
**Z3616**	pH7m24GW2	Pro35S:nGFP-FRS7
**Z3624**	pK7m24GW2	Pro35S:cGFP-FRS7
**Z2028**	pDONR207	FRS12 (STOP)
**Z2050**	pDONR207	FRS12 (NO STOP)
**Z2054**	pK7m34GW	FRS12 (NO STOP)
**Z2161**	pK7m34GW	Pro35S:FRS12-GR
**Z2587**	pFAST-R05	FRS12
**Z3205**	pENL4R1	ProFRS12 (0-585bp)
**Z3215**	pK8m34GW-FAST	35S:FRS12-3HA
**Z3615**	pH7m24GW2	Pro35S:nGFP-FRS12
**Z3623**	pK7m24GW2	Pro35S:cGFP-FRS12
**Z3224**	pmK7S*NFm14GW	ProFRS12(0-585)
**Z3439**	pK8m34-Fast	ProFRS12:FRS12:3HA
**Z4074**	pm42GW7	ProFRS12:LUC
**Z2891**	pENTR223.1-Sfi	HON4 (STOP)
**Z2888**	pENTR223.1-Sfi	AHL14 (STOP)
**Z3691**	pK7m24GW2	Pro35S:cGFP-HON4 (STOP)
**Z3695**	pK7m24GW2	Pro35S:cGFP-AHL14 (STOP)
**Z4274**	pDONR207	min35S-ProPIF4 (-3000bp)
**Z4276**	pGWLUC	min35S-ProPIF4 (-3000bp)
**Z3928**	pDONR207	ProGI (-2500bp)
**Z3974**	pGWLUC	ProGI (-2500bp)
**Z4129**	pMR217	FRS12-14
**Z4130**	pMR218	FRS7-196
**Z4189**	pDE-Cas9-Km	FRS7+12

Supplementary Table 6. Information about total read counts and mapped reads in the TChAP-Seq experiment.

Library	Total reads	QC Filtered reads	Mapped reads
**IC-2032_FRS12_HBH_N1_lib36166_2072_7_1**	13786144	8663414	8663359
**IC-2032_FRS12_HBH_N2_lib36167_2072_7_1**	17012434	11655407	11655362
**IC-2043_NLS_GFP_HBH_N2_lib41958_2328_8_1**	9305516	2151726	2151701

Supplementary Table 7. CRISPR-OR sgRNA parameters.

Gene	guide sequence + *PAM*	Specificity[1]	Efficiency[2]
FRS7	GAGCACAACCATGACCTTGG*AGG*	97	92
FRS12	GTAGGAGCTGGAGCTCTCGA*AGG*	99	86

[1]Specificity score according to Lei et al., 2014[45] (0-100).
[2]Efficiency scores according to Chari et al., 2015[46] (0-100).
